# 147. Opportunities for Improving Urine Culturing Practices in U.S. Acute Care Hospitals, 2017-2020

**DOI:** 10.1093/ofid/ofad500.220

**Published:** 2023-11-27

**Authors:** Nyawung Asonganyi, Sophia V Kazakova, James Baggs, Kelly M Hatfield, Sujan Reddy, Scott Fridkin, Joseph D Lutgring

**Affiliations:** Emory University Rolins School of Public Health, Atlanta, Georgia; CDC, atlanta, Georgia; CDC, atlanta, Georgia; Centers for Disease Control and Prevention, Atlanta, Georgia; CDC, atlanta, Georgia; Georgia Emerging Infections Program, Decatur, GA; Emory University School of Medicine, Atlanta, GA, Atlanta, Georgia; Division of Healthcare Quality Promotion, Centers for Disease Control and Prevention, Atlanta, GA

## Abstract

**Background:**

Inappropriate urine cultures (UCs) lead to misdiagnosis and unnecessary antibiotic use and can impact surveillance. The absence of pyuria on a urinalysis (UA) has a high negative predictive value for urinary tract infections (UTIs) in certain settings and can reduce unnecessary UCs. UCs performed without UA or with a negative UA indicate opportunities for diagnostic stewardship. Understanding facility variability and patient characteristics associated with inappropriate UCs can inform improvements in acute care hospitals (ACHs).

**Methods:**

Using clinical microbiology data from 279 ACHs reporting UC and UA data to the PINC AI Healthcare Database, we assessed appropriateness of the first UC collected during adult hospitalizations, excluding pregnant and urologic procedure patients identified using ICD-10-CM diagnosis codes. Each UC was categorized as follows: (1) UC with at least 1 positive UA (WBC ≥10/HPF, presence of leukocyte esterase or nitrite) 24 hours before/after UC (UA+ UC), (2) UC with negative UA in 24 hours (UA- UC), and (3) UC without UA in 24 hours (no-UA UC). Overall percentages of UC categories were described by year and patient characteristics. Monthly facility-specific percentages of UCs were calculated, and facility-level variability was described by medians, quartiles 1 and 3 (Q1:Q3).

**Results:**

1.2 million incident UCs were included. Of those, 65% were UA+, 21% UA-, and 14% no-UA UCs. From 2017 to 2020, UA+ UCs increased from 60.5% to 68.2%, no-UA UCs decreased from 19.3% to 10.5%, and UA- UCs stayed at 21%. UA- and no-UA UCs were more common among male, younger age, and Medicaid patients (Figure 1). No-UA UCs were more commonly obtained on day 4 or later. The median monthly facility-level percentages of UA+, UA-, no-UA UCs were 75 (Q1:Q3, 55.6-86.5), 15 (Q1:Q3, 6.5-28.6), and 5.3 (Q1:Q3, 2.1-10.5), respectively (Figure 2).

**Figure:**
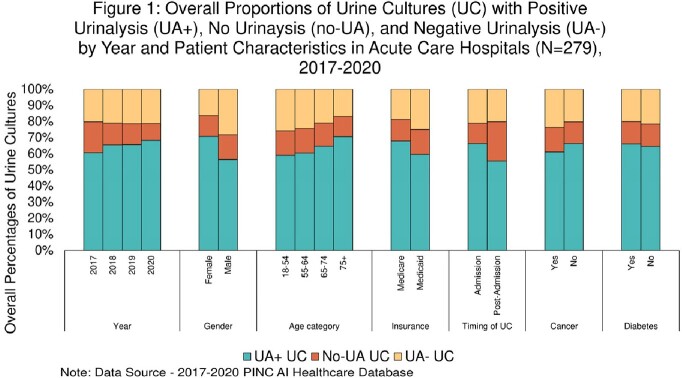

Overall Percentages of Urine Cultures

Variability in Hospital Monthly Percentages of Urine Cultures (UC) in Each Category, 2017-2020, N hospital-months = 9,863

**Figure:**
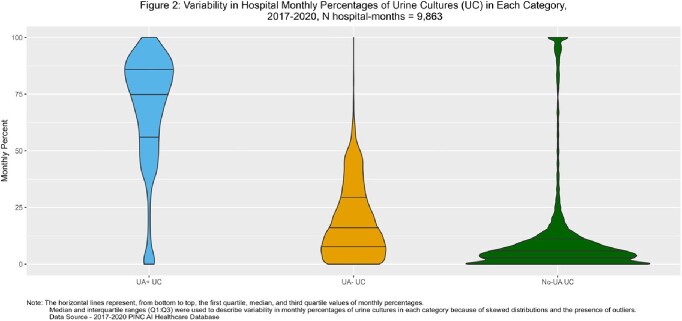

Hospital-level Variability

**Conclusion:**

During the study period, urine culturing practices improved; however, 35% of incident UCs had no UA or negative UA. The high proportion of these UCs and facility-level variability of the monthly proportions call for diagnostic stewardship to identify and address causes of the potentially suboptimal UC practices. This will support the efforts to decrease UTI overdiagnosis and antibiotic overuse.

**Disclosures:**

**All Authors**: No reported disclosures

